# Clinical EFT for individuals with visual impairment: effects on psychological wellbeing and subjective visual functioning

**DOI:** 10.1080/21642850.2026.2699488

**Published:** 2026-07-07

**Authors:** Peta Stapleton, Christine Roil

**Affiliations:** a School of Psychology, Bond University, University Drive, Robina, Queensland, Australia

**Keywords:** Vision impairment, emotional freedom techniques, anxiety, anger, depression

## Abstract

**Background:**

Visual impairment (VI) affects over 2.2 billion people globally and is often associated with heightened levels of anxiety and depression, adversely impacting quality of life. Despite increasing prevalence and associated psychosocial burden, effective psychosocial interventions in this population remains limited. This study examines the efficacy of an eight-week online Clinical Emotional Freedom Techniques (EFT) program in improving psychological wellbeing (anger, anxiety and depression) and perceived subjective visual functioning in individuals with self-reported VI.

**Method:**

Employing a randomised controlled trial design, 588 participants were assigned to either an EFT intervention (*n* = 321) or waitlist control group (*n* = 267). Outcomes were assessed at baseline, post-intervention and three- and six-month follow up.

**Results:**

Per-protocol results indicated significant improvements in the EFT group compared to the waitlist control group across psychological outcomes and vision functioning. Repeated measures ANOVA revealed significant Time × Allocation interactions for anger, anxiety, depression, and vision functioning (all *p* < .001), indicating differential change over time between groups. Within the EFT group, significant reductions in anger, anxiety, and depression were observed from pre- to post-intervention, with these improvements maintained at three- and six-month follow-ups (all *p* < .05). In contrast, the waitlist group showed no comparable improvements over the same period. Vision functioning significantly improved in the EFT group relative to the waitlist group, with a significant interaction effect (*p* < .001), although no overall main effect of time was observed. Intention-to-treat analyses using mixed-effects models confirmed the primary findings, with significant improvements in anxiety, depression, anger, and vision functioning relative to the waitlist control condition (all *p* < .001), and treatment gains were maintained at 3- and 6-month follow-up.

**Conclusions:**

These findings suggest that the online Clinical EFT intervention was associated with sustained improvements in psychological distress and vision-related functioning compared to waitlist control. Limitations and future directions are discussed.

Vision, our most dominant sense, plays a critical role in how we perceive and interact with the world. Globally, over 2.2 billion people experience some form of near or distance vision impairment (VI; World Health Organisation, 2019), and this number is expected to rise due to urbanisation and an aging population (Burton et al., 2021). VI encompasses a range of conditions affecting visual functions, varying in onset, severity, and cause. It is typically assessed through visual acuity, field of vision, contrast sensitivity, and colour vision, with impairment ranging from mild to severe (World Health Organisation, 2019).

VI has a profound psychosocial impact and is consistently associated with poorer quality of life (QoL) and wellbeing compared to sighted peers (Crews et al., 2017; Lundeen et al., 2022; Simning et al., 2019; van der Aa et al., 2015; van Nispen et al., 2016; Xiang et al., 2020). Extensive research links impaired vision to elevated levels of depression, anxiety (Brunes & Heir, 2020; Frank et al., 2019; Heesterbeek et al., 2017; Loprinzi, 2014; Simning et al., 2019; van der Aa et al., 2015), and psychological stress (Lundeen et al., 2022), as well as increased loneliness and social isolation (Brunes et al., 2019; Mick et al., 2018), which are known risk factors for depression (Ge et al., 2017; Holt-Lunstad et al., 2015). In the present study, subjective visual functioning refers specifically to self-reported visual ability and vision-related QoL. This construct captures perceived functional vision across daily activities (e.g. near and distance tasks, social functioning, role limitations), rather than objective clinical measures of visual acuity.

Emerging evidence suggests the relationship between VI and psychological wellbeing is related. Longitudinal studies indicate that vision loss increases the risk of depressive symptoms, while baseline depression predicts a higher likelihood of subsequent visual decline (Carrière et al., 2013). Similar effects have been observed in conditions such as diabetic retinopathy, where depression predicts disease incidence and progression (Khoo et al., 2019). Reciprocal associations between self-reported VI and depressive and anxiety symptoms have also been demonstrated in older adults (Frank et al., 2019). Proposed mechanisms include reduced self-care, medication-related risks, and shared neurophysiological pathways (Carrière et al., 2013), with stress potentially reinforcing this cycle by disrupting vascular regulation and increasing sympathetic nervous system activity (Sabel et al., 2018). Additionally, individuals with VI have reported feelings of anger in response to poorer QoL including reduced social engagement and increased financial burden (Brown & Barrett, 2011; Nyman et al., 2012; Teitelman & Copolillo, 2005), and evidence suggests that prolonged anger can negatively impact mental and physical health (Cassiello-Robbins & Barlow, 2016; Rohleder, 2019), further undermining psychological wellbeing and QoL.

Evidence for the effectiveness of psychosocial interventions in VI remains limited (van der Aa et al., 2016). Early reviews of low-vision rehabilitation programmes reported improvements in vision-specific QoL and functional outcomes, but inconsistent effects on depressive symptoms and broader mental health (Binns et al., 2012; Rees et al., 2010). Interventions varied widely, including practical supports (e.g. mobility training, adaptive skills) and psychological approaches such as self-management, cognitive-behavioural strategies, and counselling, with most studies focusing on short-term outcomes. As the prevalence of VI is expected to rise, there is a clear need for innovative interventions that address both visual impairment and its psychological consequences. Targeting these broader outcomes is essential for improving overall QoL and health-related quality of life (HRQoL) in individuals with VI.

## Emotional freedom techniques (EFT)

Over the past two decades, Emotional Freedom Techniques (EFT) have gained empirical attention as a psychosomatic intervention, with growing research examining their therapeutic potential across psychological (Church et al., 2022; Clond, 2016; Nelms & Castel, 2016) and physiological outcomes (Bach and Groesbeck, 2019) in diverse populations. Meta-analyses report large reductions in anxiety (d = 1.23; Clond, 2016) and depression (d = 1.31; Nelms & Castel, 2016), with effects comparable to or exceeding controls and maintained over time. Further evidence, including a systematic review, supports EFT as an effective, evidence-based treatment for both conditions (Church et al., 2022).

**Figure 1.**
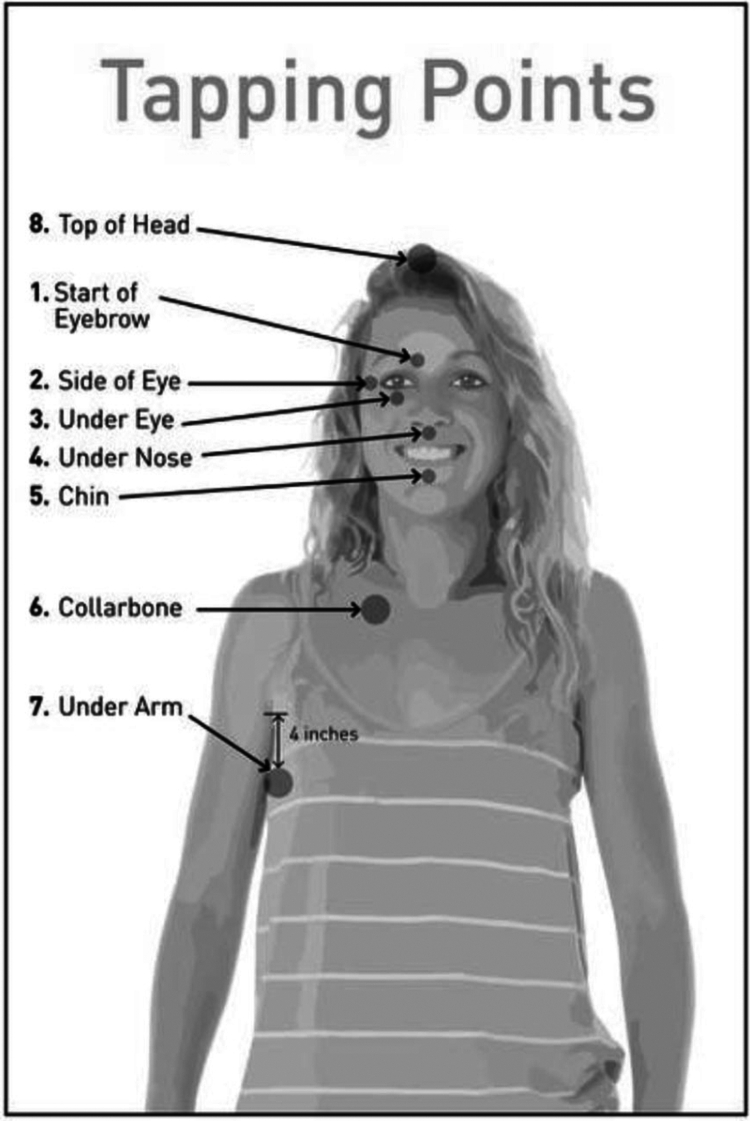
Eight Tapping Points Utilised in Clinical EFT Practice. Note: Copyright 2018 by Peta Stapleton. Reprinted with permission.

Clinical EFT is a manualised, research informed practice that integrates elements from traditional psychological interventions (cognitive behavioural therapy, CBT, exposure therapy), with a unique somatic component (Church, 2013). Specifically, a standard EFT treatment session involves tapping a specific set of eight acupoints located on the face and body (see Figure 1) while simultaneously engaging in cognitive reframing to address targeted psychological issues (Church, 2013; Stapleton & Janzen, 2025). By integrating acupoint tapping with cognitive reframing, EFT is thought to send calming signals to the amygdala and hippocampus, helping regulate the stress response (Church & Feinstein, 2017; Harper, 2012). Recent research indicates that self-paced, online Clinical EFT programmes show comparable efficacy to in-person delivery, supporting their accessibility and scalability (Church et al., 2022; Stapleton & Stewart, 2020). EFT may therefore provide a flexible, cost-effective alternative for populations facing barriers to traditional psychosocial interventions.

Evidence for Clinical EFT in vision-impaired populations is scarce, with one uncontrolled eight-week study (*n* = 120) reporting subjective improvements in vision and emotional release, though causal conclusions are limited (Look, 2007). Given EFT’s demonstrated efficacy, scalability, and low cost, further research is needed to assess its effects on psychological wellbeing and self-reported vision outcomes in this population.

## The present study

Extending Look (2007) earlier research, this study aimed to evaluate the effectiveness of EFT in improving psychological outcomes—anxiety, depression, and anger (hereafter ‘psychological outcomes’)—in individuals with VI. Specifically, we assessed an eight-week self-directed online Clinical EFT programme compared to a waitlist control, including whether any improvements were maintained over time. Prior to this, we examined correlational relationships between VI and psychological outcomes to clarify the link between visual functioning and psychological wellbeing and provide context for interpreting the primary results. Three hypotheses were proposed:

H1. There would be a negative association between self-reported visual functioning and psychological outcomes, where higher levels of anxiety, depression and anger would be associated with poorer visual functioning and conversely, poor self-reported visual functioning would be associated with poorer psychological outcomes.

H2. An eight-week online, self-directed treatment programme of Clinical EFT would result in significant decreases in psychological measures of anxiety, depression and anger compared to the waitlist control group. The post-intervention improvements were also expected to be significantly maintained for the EFT treatment group at the three- and six-month follow-up.

H3. An eight-week online, self-directed Clinical EFT programme would positively impact subjective visual functioning and vision-specific QoL compared to a waitlist control group, with improvements maintained at a three- and six-month follow-up.

## Methods

### Participants

Participants were 588 adults consisting of 45 men, 542 women and one preferring not to say. The mean age of participants ranged from 45-64 years old; however, age data were collected categorically, limiting our ability to report exact means and ranges. Inclusion criteria required participants to have moderate VI, defined as either a medical diagnosis of an eye condition or disease (e.g. cataracts, age-related macular degeneration, diabetic retinopathy, glaucoma, cytomegalovirus retinitis) or self-reported vision problems, including those with hyperopia, myopia, or other conditions requiring prescription glasses. Eligible participants were aged 18 to 80, of any gender or nationality, who could speak English, had internet access, and could independently follow a self-guided intervention. Participants were also required to provide informed consent and voluntarily commit to an eight-week trial, with no compensation provided. Individuals with complete sight loss or very low vision, which may have hindered video viewing or survey completion, were excluded from the study. Before commencement, ethical approval from the University’s Human Research Ethics Committee per the National Statement on Ethical Conduct in Human Research was obtained. The study design followed CONSORT guidelines for clinical trials (Moher et al., 2001). Additionally, the study was prospectively registered with the Australian New Zealand Clinical Trials Registry (ACTRN12624000779572).

**Figure 2.**
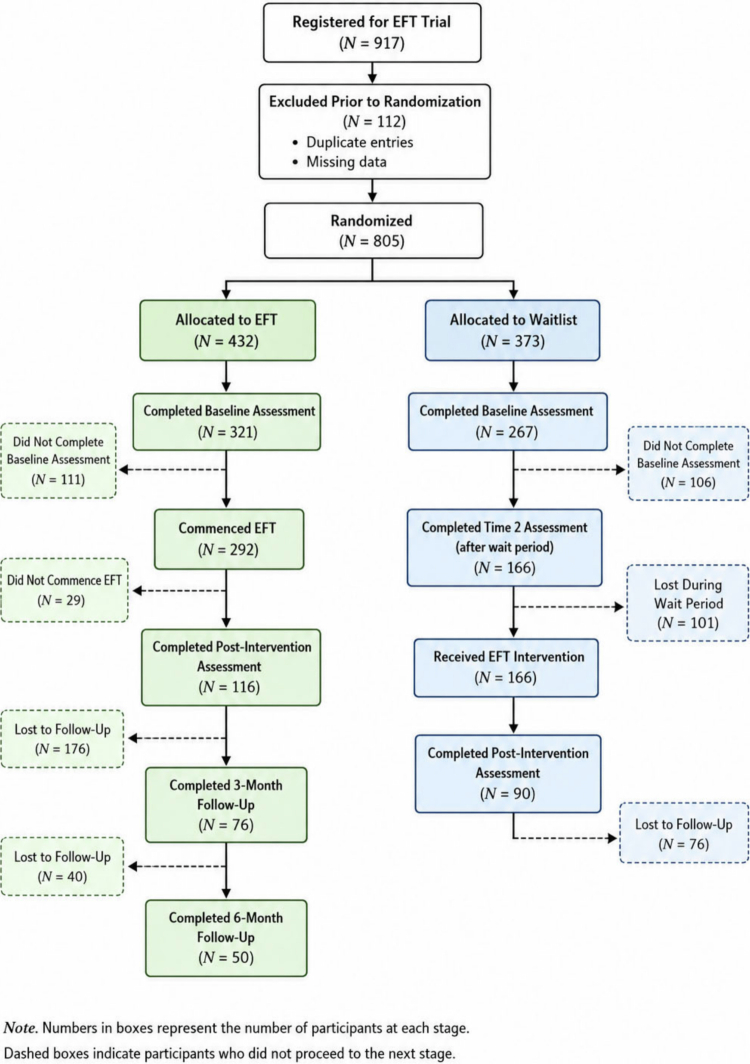
CONSORT Flow of Participants Through EFT Trial.

Recruitment for the study began in January 2024, using convenience and snowball sampling methods through the internet, forums, social media, and word-of-mouth. A total of 917 individuals responded to trial advertisements. Following the exclusion of 112 responses due to duplicate entries or missing data, 805 participants were randomised to either the EFT intervention (*n* = 432) or waitlist control condition (*n* = 373). Participants were randomly assigned via www.randomizer.org by the first author as Chief Investigator. This system employs a pseudo-random number generator, and participants were enroled on a rolling basis following expression of interest, and group allocation was assigned sequentially according to order of enrolment. Allocation was recorded manually, and no stratification or allocation concealment procedures were implemented. Of the randomised participants, 588 completed baseline assessments and provided outcome data at one or more time points (321 EFT; 267 waitlist). Intention-to-treat analyses were conducted using mixed-effects models and included all randomised participants with available data. Within the EFT condition, 321 participants completed baseline assessments (74.3%), 292 commenced the intervention (67.5%), and 116 completed post-intervention assessments (26.8%). Follow-up assessments were completed by 76 participants at 3-months and 50 participants at 6-months. Within the waitlist condition, 267 participants completed baseline assessments (71.5%), and 166 completed the second pre-treatment assessment following the wait period (44.5%). Participant flow throughout the study is presented in Figure 2.

A priori power analysis using G*Power (Version 3.1.9.6; Faul et al., 2007) determined that a minimum of 54 participants were needed for detecting a medium effect size (*d* = 0.25) with *α* = .05 (*β* = 0.95) for differences between groups (EFT treatment vs. waitlist control). Therefore, the present study exceeded the minimum sample size requirements. Demographic information for the EFT group and the waitlist control group are displayed in Table 1.

**Table 1.** Demographic Characteristics of the Participants.

Variable	EFT Group (*N* = 321) *N* (%)	Waitlist Group *(N* = 267) *N* (%)
**Gender**		
Female	294 (91.6)	248 (92.9)
Male	26 (8.1)	19 (7.1)
Prefer not to say	1 (0.3)	−
**Age**		
Young Adults (18-34)	5 (1.6)	13 (4.9)
Middle-Aged Adults (35-54)	142 (44.2)	115 (43.1)
Older Adults (55+)	174 (54.2)	139 (52.1)
**Ethnicity**		
White	299 (93.1)	242 (90.6)
Black or African American	−	2 (0.7)
Asian	10 (3.1)	12 (4.5)
Other	12 (3.7)	11 (4.1)
**Education Level**		
Less than high school	2 (0.6)	5 (1.9)
High school graduate	27 (8.4)	18 (6.7)
Undergraduate degree	85 (26.5)	82 (30.7)
Postgraduate degree	132 (41.1)	97 (36.3)
Other	18 (5.6)	12 (4.5)
Vocational college/TAFE	39 (12.1)	42 (15.7)
Doctorate	18 (5.6)	11 (4.1)
**Employment Status**		
Employed full time	114 (35.5)	92 (34.5)
Employed part time	111 (34.6)	108 (40.4)
Unemployed looking for work	11 (3.4)	6 (2.2)
Unemployed not looking for work	15 (4.7)	8 (3.0)
Retired	54 (16.8)	38 (14.2)
Student	7 (2.2)	7 (2.6)
Disabled	9 (2.8)	8 (3.0)
**Marital Status**		
Married	172 (53.6)	132 (49.4)
Widowed	11 (3.4)	8 (3)
Divorced	72 (22.4)	55 (20.6)
Separated	11 (3.4)	12 (4.5)
Never married	55 (17.1)	60 (22.5)
**Vision Impairment**		
Diagnosed with a vision impairment	287 (89.4)	234 (87.6)
Self-reported vision impairment	34 (10.6)	33 (12.4)
**Vision Impairment Diagnosis**		
Myopia	139 (43.3)	90 (33.7)
Hyperopia	49 (15.3)	51 (19.1)
Astigmatism	20 (6.2)	20 (7.5)
Presbyopia	8 (2.5)	6 (2.2)
Cataracts	9 (2.8)	5 (1.9)
Age Related Conditions	8 (2.5)	9 (3.4)
Other/Complex	17 (5.3)	25 (9.4)
Vision Correction	29 (9.0)	28 (10.5)
General Vision Issues	2 (0.6)	1 (0.4)
Not Specified	40 (12.5)	32 (10)

Note: *n = number of cases. Age data were consolidated into broader categories, and participants' VI diagnoses were categorised after reviewing the data for common responses.*

### Materials

A pretrial survey comprised demographic items (age, gender, marital and employment status, ethnicity, education, and VI diagnosis). Pre- and post-intervention measures assessed participants' self-reported psychological and vision outcomes.

***Hospital Anxiety and Depression Scale*** **(HADS; Zigmond & Snaith, 1983**): A 14-item self-report measure of anxiety (HADS-A) and depression (HADS-D), scored on a 0–3 scale, with subscale totals (0–21) and scores > 8 indicating mild–severe symptoms. It demonstrates good reliability and validity (Bjelland et al., 2002), with strong internal consistency in this sample (*α* = .85–.80).

***Novaco Anger Scale and Provocation Inventory*** **(NAS-PIE; Novaco, 2003):** The Provocation Inventory (25 items) was used to assess anger triggers across five domains, scored on a 0–4 scale (total 0–100), with higher scores indicating greater anger. The scale shows strong reliability and validity (Culhane & Morera, 2010; Hornsveld et al., 2011), with excellent internal consistency in this sample (*α* = .94).

***National Eye Institute Visual Functioning Questionnaire*** **(NEI-VFQ-25; Mangione et al., 2001):** A 25-item measure of vision-related functioning and quality of life across 11 domains, scored from 0–100, with higher scores reflecting better functioning. It demonstrates strong reliability and validity (Mangione et al., 2001; Orr et al., 2011), with excellent internal consistency in this sample (*α* = .92).

### Treatments

The Clinical EFT online treatment was a self-paced eight-week programme delivered online via the secure platform Kajabi (https://kajabi.com). The original audio files used by Look (2007), a trained EFT practitioner, were re-recorded by the original presenter (Dr Carol Look) in video format for this study. Table 2 presents an overview of the weekly themes addressed throughout the 8-week EFT programme.

**Table 2.** Overview of EFT Intervention: Sessions and Themes.

Session	Theme
Week 0	Introduction to EFT Tapping for Vision Issues
Week 1	Tapping for Resistance
Week 2	Tapping for Fear
Week 3	Tapping for Guilt
Week 4	Tapping for Anger
Week 5	Tapping for Anxiety
Week 6	Tapping for Beliefs
Week 7	Tapping for Resentment
Week 8	Tapping for Removing Blocks

All sessions followed the standardised protocols outlined in *The EFT Manual* (Church, 2013) and lasted approximately six minutes, during which participants were guided through three rounds of EFT tapping. Each round required participants to recite a ‘setup statement’ whilst tapping an acupoint on the side of the hand. An example setup statement from module one was, ‘Even though I have this resistance about improving my eyesight, I deeply and completely love and accept myself anyway’. Participants then stimulated a pre-established set of eight acupuncture points (see Figure 1) by tapping them with their fingertips whilst repeating ‘reminder phrases’. An example of a reminder phrase from module one was, ‘This resistance to improving my eyesight’. In between each round, participants were required to rate the intensity of the feeling or memory being experienced using a Subjective Units of Distress (SUD) rating (Wolpe, 1973), an 11-point Likert scale ranging from 0 (*no distress*) to 10 (*maximum distress*), and to note any changes in their ratings. At the end of each session, participants were encouraged to repeat as many rounds as needed and tap daily, with or without the video, until they observed a reduction in their SUD rating.

Those in the waitlist group received no treatment during the initial study period. Following the completion of the study, individuals in the waitlist group were offered the opportunity to engage in the EFT treatment, allowing for equitable access to the intervention.

### Procedure

Individuals expressing interest in the study were emailed a link to an online presurvey containing an explanatory statement, consent form, and demographic questions. Following consent, participants completed baseline measures assessing visual functioning and psychological outcomes before being randomly allocated to either the EFT intervention or a waitlist control group. Group allocation was communicated via email.

Participants assigned to the EFT intervention group were granted online access to an eight‑week, self‑paced programme, delivered between February and April 2024. Assessments were conducted at baseline (January 2024), post‑treatment (April 2024), and at three‑ and six‑month follow‑up (July and October 2024). Weekly reminder emails provided access to each module, with content released sequentially to standardise treatment exposure; participants could revisit prior modules but were unable to access future sessions in advance. Each session included an accompanying worksheet, and participants were encouraged to review and repeat recorded materials. Optional peer support was available via a private Facebook group moderated by the chief investigator (first author), which was utilised by approximately half of participants (51%).

The waitlist control group followed the same EFT intervention protocol after a delay, completing treatment between April and June 2024. Data were collected at baseline (January 2024), a second pre‑treatment time point aligned with the intervention group’s post‑treatment assessment (April 2024), immediately post‑treatment (June 2024). Identifiable information was collected solely for platform access and data linkage, with all analyses conducted on de‑identified data.

### Data diagnostics

Data was imported from Qualtrics XM (Qualtrics, 2024). Data screening and statistical analyses were conducted using IBM SPSS Statistics (version 29.0.2.0; IBM Corp, 2023). Data was initially visually screened for keystroke errors, and participants with considerable missing data were removed for per-protocol analysis (however intention-to-treat analysis was also completed—see below). Participants were allocated a unique identifier, and all previous identifying details were removed. Statistical significance was evaluated at *α* = .05. Effect sizes were reported as Pearson’s correlation coefficient (r) for correlational analyses and partial eta squared (η^2^ *p*) for ANOVAs. Partial eta squared values of .01, .06, and .14 were interpreted as small, medium, and large effects, respectively (Cohen, 1988). All pairwise comparisons used Bonferroni adjustment. Results reported were derived using a per-protocol analysis that employed listwise deletion to address missing data; therefore, participants were required to complete both pre- and post-survey data for inclusion in the complete case analysis. Baseline characteristics of the EFT treatment condition and the waitlist control group were compared using the Chi-Square Test of Independence and there were no differences in demographic characteristics, suggesting balanced allocation. Participants in the waitlist group were assessed twice during the control waiting period and four paired sample t-tests were used to compare the outcome variables between the two time points. The results showed no significant differences, suggesting that participants in the waitlist group experienced no notable changes in outcome variables during the waiting period. To test each of the hypotheses, three correlation analyses were performed to explore the associations among each outcome variable. Following this, four 2 × 2 mixed-model analyses of variance (ANOVA) were conducted, followed by four one-way repeated measures ANOVAs to investigate any immediate and sustained effects of the EFT treatment between the EFT and waitlist groups.

## Results

Assumption testing included descriptive statistics and normality cheques (Shapiro-Wilk, histograms, Q–Q plots), with minor deviations observed but not considered problematic given sample characteristics and ANOVA robustness (Allen et al., 2023; Field, 2018; Mangione et al., 2001; Tabachnick & Fidell, 2019; Tomitaka, 2020). HADS-D and NEI-VFQ-25 showed expected skew patterns, and one extreme NAS outlier (ID = 516) was identified during data screening and removed due to its potential to disproportionately influence analyses. Sensitivity cheques indicated that inclusion of this case did not alter the pattern or significance of results. Table 3 overviews the descriptive statistics.

**Table 3.** Descriptive Statistics for Pre-Survey Outcome Variables (*n* = 588).

Outcome	Mean	SD	Min	Max	Skewness	Kurtosis
Anger	44.83	16.29	1	95	.42	.06
Anxiety	8.49	4.03	0	19	.24	−.53
Depression	5.36	3.48	0	18	.56	−.083
Visual Function & Vision Related QoL	82.38	13.13	21	100	−1.37	2.11

### Main analysis

The main analyses are presented in the following order. First, we examined the relationship between psychological and vision outcomes (H1). Second, we investigated the immediate effect of EFT treatment on psychological outcomes, comparing pre- and post-treatment results between the EFT and waitlist groups. Next, we investigated the sustained effect of treatment by comparing psychological outcomes at follow-up with those at pre- and post-treatment (H2). Lastly, we investigated the immediate effects of EFT treatment on vision outcomes, comparing pre- and post-treatment results between the EFT and waitlist groups and the sustained effects of treatment over time by comparing vision outcomes at follow-up with those at pre- and post-treatment (H3). Reported outcomes are pre protocol analyses, but intention-to-treat analyses were conducted using linear mixed-effects models with restricted maximum likelihood (LMMs). All randomised participants were included in the analyses regardless of treatment completion. Models included fixed effects for group (EFT vs. waitlist), time, and the Group × Time interaction, with participant entered as a random effect. Linear mixed models were selected because they accommodate incomplete repeated-measures data without requiring ad hoc imputation procedures and provide valid estimates when data are missing at random. Estimated marginal means and 95% confidence intervals were calculated for all outcomes. Statistical significance was set at *p* < .05 (two-tailed).

#### Hypothesis one

Initial bivariate correlations indicated significant negative associations between subjective visual functioning (NEI VFQ‑25) and anger (r = −.21, *p* < .001), anxiety (r = −.32, *p* < .001), and depression (r = −.35, *p* < .001), such that higher psychological distress was associated with poorer self-reported visual functioning (see Table 4). Cohen, (1988) effect size ranged between –.22 to −.36.

**Table 4.** Correlation Analysis of NAS, HADS Anxiety and Depression, and NEI VFQ-25 Composite Scores.

		NAS Presurvey Total	HADS_Anxiety Presurvey Total	HADS_Depression Presurvey Total
NEI VFQ-25 Presurvey Total	Pearson Correlation	−.210**	−.324**	−.352^*^
	Sig. (2-tailed)	<.001	<.001	<.001
	N	584	584	584

Note: ** = Correlation is significant at the 0.01 level (2-tailed). NEI VFQ-25 = National Eye Institute Visual Functioning Questionnaire-25, NAS = Novaco Anger Scale, HADS = Hospital Anxiety and Depression Scale.

#### Hypothesis two

To examine the immediate effect of the EFT intervention on the psychological variables of interest compared to the waitlist control group, three 2 × 2 mixed-model ANOVAs were conducted. Sample sizes varied across analyses due to listwise deletion of missing data in per-protocol analyses and are reported within each table. Accordingly, the number of participants included differed slightly across outcome variables. Intention-to-treat analyses, which included all available data, are reported separately in the Supplementary Materials and overviewed below. Descriptive statistics for the groups are presented in Table 5, and results of the mixed ANOVAs are presented in Table 6. To examine the sustained effect of the EFT intervention on the variables of interest, three one-way repeated measures ANOVAs were conducted. Time was the repeated measures variable (pre-test vs. follow-up and post-test vs. follow-up). Descriptive statistics for each time phase are presented in Table 7, and results of the one-way repeated measures ANOVAs are presented in Table 8. All assumption testing was met unless otherwise stated. Where applicable, the Greenhouse–Geisser correction was used to adjust for violations of sphericity, and corrected degrees of freedom are reported.

**Table 5.** Descriptive Statistics for the Immediate Effects of Treatment on Psychological Outcomes.

			Pre		Post	
Outcome	Group	*n*	Mean	SD	Mean	SD
Anger	EFT	123	45.38	16.17	37.36	14.56
	Waitlist	156	43.11	15.58	43.04	15.80
Anxiety	EFT	123	8.62	3.97	6.64	3.70
	Waitlist	157	8.38	3.95	8.15	3.91
Depression	EFT	123	5.60	3.34	4.26	3.01
	Waitlist	157	5.68	3.19	5.68	3.43

Note: The differences in sample sizes (*n*) for each measure are due to listwise deletion, where cases with missing data were removed from the analysis.

**Table 6.** ANOVA Results and Mean Differences Between EFT and Waitlist Groups for Psychological Outcome Variables.

								95% Confidence Interval	
Outcome	df effect	df error	F	p	η2	*M* Diff.	Std. Error	Lower Bound	Upper Bound
Anger	1	277	31.81	<.001	.10	−8.02*	1.05	−9.30	−2.06
Anxiety	1	278	29.33	<.001	.11	−1.51*	.47	−2.42	−.60
Depression	1	278	32.02	<.001	.13	−1.42*	.39	−2.19	−.65

Note: *p < 0.001. Mean differences and confidence intervals represent pairwise comparisons between EFT and Waitlist groups. Only the interaction effect is reported as the primary hypothesis examined the effectiveness of EFT relative to the waitlist control group.

Significant Time × Group interaction effects were observed across all psychological outcomes (Table 6), indicating that changes over time differed between the EFT and waitlist groups. Participants in the EFT condition demonstrated significant reductions in anger, anxiety, and depression from pre- to post-intervention, whereas the waitlist group showed minimal change across the same period. Baseline differences between groups were non-significant for all outcomes. The magnitude and direction of effects were consistent across measures, suggesting that improvements in psychological distress were attributable to the EFT intervention rather than natural change over time.

Within the EFT group, reductions from pre- to post-intervention were significant across all outcomes and were maintained at follow-up (see Tables 7–8). No comparable improvements were observed in the waitlist condition.

**Table 7.** Descriptive Statistics for the Sustained Effects of EFT Treatment on Psychological Outcomes.

	Pre (*n*=32)		Post (*n*=32)		3-M FU (*n*=32)		6-M FU (*n*=32)	
Outcome	Mean	SD	Mean	SD	Mean	SD	Mean	SD
Anger	38.47	14.67	32.34	12.05	33.69	11.83	31.94	12.69
Anxiety	7.25	3.68	6.06	4.32	5.84	4.05	5.81	3.53
Depression	5.41	3.34	3.69	2.71	4.03	3.38	4.25	2.82

Note: *n* = number of cases.

**Table 8.** Repeated Measures ANOVA and Pairwise Mean Differences for the EFT Group: Pre, Post, and Six-Month Follow-Up.

Outcome	*df* (effect)	*df* (error)	*F*	*p*	η^2^p		Mean Difference	SE	95% CI Lower	95% CI Upper
**Anger**	2.33	72.28	5.40	.004	.148					
						Pre—Post	6.13*	2.09	1.87	10.39
						Pre—FU	6.53*	2.14	2.18	10.89
						Post—FU	0.41	1.35	-2.36	3.17
**Anxiety**	2.74	84.99	4.81	.005	.134					
						Pre—Post	1.19*	0.52	0.12	2.26
						Pre—FU	1.44*	0.43	0.57	2.31
						Post—FU	0.25	0.41	-0.59	1.09
**Depression**	2.62	81.22	6.04	.002	.163					
						Pre—Post	1.72*	0.45	0.79	2.65
						Pre—FU	1.16*	0.42	0.30	2.02
						Post—FU	0.56	0.36	-0.18	1.30

Note: df = degrees of freedom; F values for the Greenhouse–Geisser corrected repeated measures ANOVA are reported. η^2^ *p* = partial eta squared. FU = 6-month follow-up. Mean differences are based on estimated marginal means (least significant difference, unadjusted). *p* values are two-tailed. *p* < .05 is indicated by *. CI = confidence interval. Negative values indicate reductions over time depending on subtraction order.

#### Hypothesis three

Descriptive patterns showed that the EFT group improved from baseline to post-intervention, whereas the waitlist group remained relatively stable (Table 9). A 2 × 2 mixed-model ANOVA indicated no significant main effect of time, but a significant Time × Allocation interaction (*p* < .001; Table 10), demonstrating differential change between groups. Improvements in the EFT group were maintained at 3- and 6-month follow-up assessments (Table 11), indicating sustained gains in subjective visual functioning.

**Table 9.** Descriptive Statistics for the Immediate Effects of Treatment on Composite Vision Outcome.

			Pre		Post	
Outcome	Group	*n*	Mean	SD	Mean	SD
Visual Function & Vision Related QoL	EFT	123	83.59	11.77	88.07	10.06
	Waitlist	156	81.94	13.65	81.71	14.16

**Table 10.** ANOVA results and mean differences between eft and waitlist groups for composite vision outcome.

Outcome	*df* (effect)	*df* (error)	*F*	*p*	η^2^p	Comparison	Mean Difference	SE	95% CI Lower	95% CI Upper
Vision Functioning (VFQ)	2.35	72.74	0.29	.781	.009	Time (within-subjects)				
	1.00	277	21.08	<.001	.071	Time × Allocation				
						Pre (Waitlist)	81.94	13.65	—	—
						Pre (EFT)	83.59	11.77	—	—
						Post (Waitlist)	81.71	14.16	—	—
						Post (EFT)	88.07	10.06	—	—

Note: *p* < .001. Mean differences and confidence intervals refer to pairwise comparisons between EFT and waitlist groups. The Time × Allocation interaction is reported as the primary test of the study hypothesis, as it examines differential change over time between groups.

**Table 11.** Descriptive statistics for the sustained effects of eft treatment on vision outcome.

	Pre (*n*=32)		Post (*n*=32)		3 M Follow Up (*n*=32)		6 M Follow Up (*n*=32)	
Outcome	Mean	SD	Mean	SD	Mean	SD	Mean	SD
Visual Function & Vision Related QoL	88.25	10.66	89.06	10.78	88.65	12.76	89.26	10.65

Note: *n* = number of cases.

### Intention-to-treat analysis

Intention-to-treat analyses were conducted using linear mixed-effects models estimated with restricted maximum likelihood (Table 12). Linear mixed-effects models estimated using restricted maximum likelihood were employed for this, as this approach uses all available observations and provides valid estimates under the assumption that data are Missing at Random (MAR). Significant Group × Time interactions were observed across all outcomes during the randomised phase, indicating greater improvements among participants receiving EFT relative to waitlist controls (all *p* < .001). Longitudinal follow-up analyses further demonstrated significant improvements across all outcomes following treatment, with gains maintained at both 3- and 6-month follow-up assessments and no evidence of deterioration over time.

**Table 12.** Intention-to-Treat Mixed-Effects Analyses Comparing EFT and Waitlist During the Randomised Phase and Follow-Up.

Outcome	Randomised Phase ITT: Waitlist Baseline	Waitlist 8 Weeks	EFT Baseline	EFT 8 Weeks	Group × Time F(df)	p	Follow-Up ITT: Pre-Treatment	Post-Treatment	3 Months	6 Months	Time F(df)	p
HADS Anxiety	8.70	8.40	8.31	6.34	26.94 (1,307.76)	<.001	8.28	6.65	6.80	6.71	27.36 (3,358.74)	<.001
HADS Depression	5.42	5.73	5.31	3.93	33.47 (1,304.93)	<.001	5.45	3.96	4.31	4.40	27.81 (3,344.38)	<.001
Novaco Anger Scale	44.75	44.24	44.90	36.50	32.45 (1,310.81)	<.001	44.36	36.61	36.46	36.70	36.21 (3,367.01)	<.001
VFQ Composite	81.82	81.61	82.86	87.77	25.05 (1,300.90)	<.001	82.40	87.30	87.82	87.21	27.71 (3,343.17)	<.001

Note: Values are estimated marginal means from mixed-effects models estimated using restricted maximum likelihood. Randomised phase analyses compared participants receiving EFT with waitlist controls over the initial 8-week period. Follow-up analyses pooled participants following receipt of EFT and examined maintenance of treatment gains through 6 months.

## Discussion

The current study sought to evaluate the effects of an eight-week online Clinical EFT programme on psychological outcomes and perceived subjective visual functioning in individuals with visual impairment. The findings indicate that participation in the EFT programme was associated with improvements in anger, anxiety, depression, and perceived visual functioning, relative to a waitlist control group. However, results should be interpreted as evidence of association rather than definitive treatment efficacy at the population level. Supporting our first hypothesis, we found a significant association between psychological outcomes and visual functioning, whereby higher levels of anxiety, depression, and anger were associated with poorer visual functioning, and poorer self-reported visual functioning was associated with worse psychological outcomes. This finding reinforces the interrelated nature of mental health and vision, consistent with previous literature (Carrière et al., 2013; Frank et al., 2019; Khoo et al., 2019). The observed association suggests that changes in one domain may influence the other, highlighting the importance of considering both psychological and visual factors in the management of individuals with VI.

Building on this relationship, supporting our second hypothesis, the eight-week self-directed online Clinical EFT programme demonstrated significant efficacy in improving psychological outcomes. Participants in the EFT group showed significant reductions in anger, anxiety, and depression from pre- to post-intervention, with these improvements maintained at three- and six-month follow-ups relative to the waitlist group. Baseline comparisons indicated no significant differences between groups, suggesting that observed improvements can be attributed to the intervention. Notably, anxiety scores in the EFT group moved from the borderline abnormal range into the normal range following the intervention, whereas the waitlist group showed no comparable change. These findings are consistent with prior research demonstrating the efficacy of EFT in reducing anxiety and depression (Church et al., 2022; Clond, 2016; Nelms & Castel, 2016), and are in line with theoretical links between anxiety, depression, and anger as interrelated emotional states (Cassiello-Robbins & Barlow, 2016; Eysenck & Fajkowska, 2018). Importantly, these improvements were sustained over time, indicating enduring treatment effects.

In support of our third hypothesis, the EFT intervention was also associated with perceived improvements in subjective visual functioning and vision-related QoL relative to the waitlist group. While there was no significant main effect of time, a significant Time × Allocation interaction indicated that changes in visual functioning differed between groups, with the EFT group demonstrating improvements from baseline to post-intervention that were maintained across follow-up assessments. By post-test, participants in the EFT group reported perceived higher NEI VFQ-25 composite scores compared to the waitlist group, consistent with findings from Look (2007). Given the established relationship between visual impairment and psychological wellbeing (Carrière et al., 2013; Frank et al., 2019; Khoo et al., 2019), it is plausible that reductions in psychological distress contributed to improvements in perceived visual functioning. As symptoms of anger, anxiety, and depression decreased, participants may have experienced more favourable perceptions of their visual functioning, reflecting the possible interconnected nature of mental and visual health. It should also be noted that approximately half of participants engaged with an optional peer-support Facebook group, which may have contributed to improvements through increased perceived social support or engagement, independent of the EFT intervention.

### Limitations

Several limitations should be considered when interpreting the findings of this study. Substantial attrition observed at follow-up remains a limitation. Beyond reducing statistical power, this level of dropout introduces the potential for attrition bias. Participants who completed the intervention may differ systematically from those who withdrew (e.g. in motivation, engagement, or responsiveness to treatment), which may result in overestimation of treatment effects. Although intention-to-treat analyses using mixed-effects models were employed to mitigate bias associated with missing data, these approaches assume data are missing at random and cannot fully account for systematic differences between completers and non-completers. As such, the findings may reflect a more engaged or treatment-responsive subset of participants. This limits the generalisability of results to the broader population of individuals with visual impairment, and future studies should prioritise strategies to improve retention and examine predictors of dropout.

The sample was also predominantly female (90%), which is consistent with participation trends in online psychosocial and complementary therapy research. Although randomisation ensured comparable gender distribution across conditions, this imbalance limits the generalisability of the results to the broader population of individuals with visual impairment. Future studies should prioritise targeted recruitment strategies to increase representation of underrepresented genders.

Second, all outcomes were assessed using self‑report measures. While validated instruments such as the NEI‑VFQ‑25 are widely used to evaluate subjective visual functioning and vision‑related quality of life, reliance on self‑report limits conclusions about objective visual change. An objective acuity measure (Snellen) was planned; however, the fully remote nature of the trial prevented standardised administration (e.g. consistent viewing distance, screen calibration, lighting), rendered these data invalid for analysis. Including them would have risked misinterpretation. As the NEI‑VFQ‑25 assesses subjective functional vision rather than clinical acuity, the findings should be interpreted accordingly. Future studies should employ clinician‑administered or digitally standardised acuity assessments to ensure reliable objective measurement. Further, only the NEI‑VFQ‑25 composite score was analysed due to project constraints, preventing examination of individual functional domains. Future research should incorporate clinician‑administered or digitally standardised visual assessments and more granular analysis of VFQ‑25 subscales to enhance clinical interpretability.

In addition to the above considerations, the reliance on subjective self‑report introduces further methodological constraints that warrant discussion. Self‑reported outcomes are vulnerable to temporal, expectancy‑based, and psychological artefacts, particularly in longitudinal designs. As highlighted in methodological work on temporal control frameworks, distinguishing genuine intervention effects from changes that arise simply due to the passage of time, repeated measurement, or rhythmic psychological fluctuations is essential for strengthening causal inference (Dhahbi & Dergaa, 2026). Although the inclusion of a waitlist control group helps mitigate these risks, future research would benefit from incorporating more sophisticated temporal‑control designs or analytic strategies capable of isolating intervention‑specific change from inherent temporal variability.

Similarly, the use of unverified online self‑reporting reflects a broader challenge in remote behavioural and clinical research. Recent methodological commentary emphasises the need for standardised, technology‑supported, or sensor‑based objective assessments to validate subjective outcomes and reduce measurement error in decentralised trials (Dhahbi & Chamari, 2026). While such tools were beyond the scope of the present study, integrating validated digital acuity tests, calibrated remote‑assessment platforms, or clinician‑verified ophthalmic data would substantially enhance the robustness and clinical applicability of future work.

Regarding the external support offered, only 51% of participants engaged with an optional, investigator-moderated Facebook support group. While this component was included to enhance engagement and provide a sense of social connection within a fully self-directed programme, participation was not standardised and engagement was not systematically measured. As a result, differing levels of peer interaction and perceived social support may have influenced psychological outcomes, representing a potential confounding factor. Future studies should quantify engagement and consider controlling or standardising adjunct support elements to better isolate intervention effects.

Recruitment via convenience and snowball sampling may have introduced selection bias, and visual impairment status was based on self‑report rather than clinician‑verified diagnosis. The sample included individuals with both refractive errors (e.g. myopia, hyperopia) and pathological visual conditions. This broad inclusion reflects the study’s focus on subjective functional vision; however, it limits diagnostic precision and the ability to generalise findings specifically to populations with medically confirmed pathological VI. Sub‑analyses isolating pathological conditions were not feasible due to sample size constraints and attrition across follow‑up assessments. Future studies should consider stratified recruitment or narrower inclusion criteria to enable condition‑specific analyses and improve diagnostic validity.

Improvements observed in NEI‑VFQ‑25 scores should be interpreted as changes in perceived visual functioning rather than changes in ocular pathology. The NEI‑VFQ‑25 is sensitive to psychological factors such as mood, coping, and self‑efficacy, and the observed Time × Allocation interaction may reflect enhanced emotional wellbeing rather than clinical visual improvement—particularly for participants with refractive error, for whom physiological change would not be expected. Incorporating objective ophthalmic measures and stratified analyses in future research will help clarify the relationship between psychological interventions, subjective visual functioning, and clinical visual outcomes.

The study utilised a waitlist control group rather than an active comparator. While this design allows for evaluation of intervention effects relative to no treatment and controls for temporal change, it does not account for non-specific influences such as expectancy or placebo effects. As such, the extent to which observed improvements reflect specific versus non-specific treatment factors cannot be fully determined. Future research should incorporate active control conditions (e.g. behavioural activation or self-management interventions; van der Aa et al., 2016) to better isolate intervention-specific effects and strengthen causal inference.

Finally, the study was unblinded, which is typical for self-directed behavioural interventions but nonetheless introduces potential sources of bias. Participants were aware of their involvement in an intervention study, although specific hypotheses were not disclosed. The Chief Investigator first author, who managed enrolment, allocation, and intervention delivery, was not blinded to participant identity or group assignment. Outcomes were assessed via self-report without blinded assessors. Although data analysis was conducted by a researcher independent of recruitment and intervention delivery (second author), the absence of blinding may have increased the risk of expectancy and reporting biases.

## Conclusion

Despite the noted limitations, to our knowledge, this study is the first randomised controlled trial to examine the efficacy of Clinical EFT in individuals with visual impairment. The findings contribute to the growing body of evidence providing preliminary evidence supporting EFT as a potentially effective intervention for psychological outcomes. In addition, the results provide preliminary evidence that EFT may enhance perceived subjective visual functioning and vision-related QoL. The sustained improvements observed across follow-up assessments suggest that EFT may offer a cost-effective, accessible, and durable intervention for individuals with VI. However, replication in larger and more diverse samples, the inclusion of active comparator conditions, and the incorporation of objective ophthalmic measures will be important to more definitively establish the efficacy and mechanisms of the intervention. These findings also highlight the importance of integrating psychological considerations into vision care. Training eye care professionals to recognise symptoms of anxiety and depression may facilitate timely referral and support, ultimately improving mental health outcomes and overall quality of life for individuals living with visual impairment.

## Supplementary Material

Supplementary Material ITT_Analysis_Eyesight_EFT_SupplementaryCleanVersion.docx

## Data Availability

The data that support the findings of this study are available by request at https://osf.io/z9p36/overview?view_only=fb302bb9eb3048e1b72f22efc27e4054.

## References

[cit0001] Allen, P. , Bennett, K. , & Heritage, B. (2023). *SPSS statistics: A practical guide* (5th ed.). Cengage Learning.

[cit0002] Bach, D. , Groesbeck, G. , Stapleton, P. , Sims, R. , Blickheuser, K. , & Church, D. (2019). Clinical EFT (Emotional Freedom Techniques) improves multiple physiological markers of health. *Journal of Evidence-Based Integrative Medicine* , *24* , 1–12. 10.1177/2515690X18823691 PMC6381429 30777453

[cit0003] Binns, A. M. , Bunce, C. , Dickinson, C. , Harper, R. , Tudor-Edwards, R. , Woodhouse, M. , Linck, P. , Suttie, A. , Jackson, J. , Lindsay, J. , Wolffsohn, J. , Hughes, L. , & Margrain, T. H. (2012). How effective is low vision service provision? A systematic review. *Survey of Ophthalmology* , *57* (1), 34–65. 10.1016/j.survophthal.2011.06.006 22018676

[cit0004] Bjelland, I. , Dahl, A. A. , Haug, T. T. , & Neckelmann, D. (2002). The validity of the hospital anxiety and depression scale: An updated literature review. *Journal of Psychosomatic Research* , *52* (2), 69–77. 10.1016/S0022-3999(01)00296-3 11832252

[cit0005] Brown, R. L. , & Barrett, A. E. (2011). Visual impairment and quality of life among older adults: An examination of explanations for the relationship. *The Journals of Gerontology Series B, Psychological Sciences and Social Sciences* , *66* (3), 364–373. 10.1093/geronb/gbr015 21402645

[cit0006] Brunes, A. , & Heir, T. (2020). Visual impairment and depression: Age-specific prevalence, associations with vision loss, and relation to life satisfaction. *World Journal of Psychiatry* , *10* (6), 139–149. 10.5498/wjp.v10.i6.139 32742947 PMC7360524

[cit0007] Brunes, A. , B. Hansen, M. , & Heir, T. (2019). Loneliness among adults with visual impairment: prevalence, associated factors, and relationship to life satisfaction. *Health and Quality of Life Outcomes* , *17* (1), 10.1186/s12955-019-1096-y PMC6359849 30709406

[cit0008] Carrière, I. , Delcourt, C. , Daien, V. , Pérès, K. , Féart, C. , Berr, C. , Laure Ancelin, M. , & Ritchie, K. (2013). A prospective study of the bi-directional association between vision loss and depression in the elderly. *Journal of Affective Disorders* , *151* (1), 164–170. 10.1016/j.jad.2013.05.071 23787409

[cit0009] Cassiello-Robbins, C. , & Barlow, D. H. (2016). Anger: The unrecognized emotion in emotional disorders. *Clinical Psychology: Science and Practice* , *23* (1), 66–85. 10.1111/cpsp.12139

[cit0010] Church, D. (2013). *The EFT manual* (Third ed.). Energy Psychology Press.

[cit0011] Church, D. , & Feinstein, D. (2017). The manual stimulation of acupuncture points in the treatment of post-traumatic stress disorder: A review of clinical emotional freedom techniques. *Medical Acupuncture* , *29* (4), 194–205. 10.1089/acu.2017.1213 28874920 PMC5580368

[cit0012] Church, D. , Stapleton, P. , Vasudevan, A. , & O’Keefe, T. (2022). Clinical EFT as an evidence-based practice for the treatment of psychological and physiological conditions: A systematic review. *Frontiers in Psychology* , *13* . 951451. 10.3389/fpsyg.2022.951451 36438382 PMC9692186

[cit0013] Clond, M. (2016). Emotional freedom techniques for anxiety: A systematic review with meta-analysis. *The Journal of Nervous and Mental Disease* , *204* (5), 388–395. 10.1097/NMD.0000000000000483 26894319

[cit0014] Cohen, J. (1988). *Statistical power analysis for the behavioral sciences* (2nd ed.). L. Erlbaum Associates.

[cit0015] Crews, J. E. , Chou, C.-F. , Sekar, S. , & Saaddine, J. B. (2017). The prevalence of chronic conditions and poor health among people with and without vision impairment, aged ≥65 years, 2010–2014. *American Journal of Ophthalmology* , *182* , 18–30. 10.1016/j.ajo.2017.06.038 28734819

[cit0016] Culhane, S. E. , & Morera, O. F. (2010). Reliability and validity of the novaco anger scale and provocation inventory (NAS-PI) and state-trait anger expression Inventory-2 (STAXI-2) in hispanic and non-hispanic White student samples. *Hispanic Journal of Behavioral Sciences* , *32* (4), 586–606. 10.1177/0739986310381458

[cit0017] Dhahbi, W. , & Chamari, K. (2026). The algorithmic athlete: A call to standardize assessment of sensor technologies and artificial intelligence. *International Journal of Sports Physiology and Performance* , *21* (4), 505–506. 10.1123/ijspp.2025-0547. Retrieved Jun 2, 2026, from. 41493778

[cit0018] Dhahbi, W. , & Dergaa, I. (2026). Machine learning with temporal control designs for testing rhythmic specificity in chronobiology: A multivariate framework proposal for distinguishing genuine biological rhythms from temporal artifacts. *Chronobiology International* , 43, 1–8. 10.1080/07420528.2026.2650826 41920774

[cit0019] Eysenck, M. W. , & Fajkowska, M. (2018). Anxiety and depression: Toward overlapping and distinctive features. *Cognition and Emotion* , *32* (7), 1391–1400. 10.1080/02699931.2017.1330255 28608767

[cit0020] Faul, F. , Erdfelder, E. , Lang, A.-G. , & Buchner, A. (2007). GPower 3: A flexible statistical power analysis program for the social, behavioral, and biomedical sciences. *Behavior Research Methods* , *39* (2), 175–191. 10.3758/bf03193146 17695343

[cit0021] Field, A. P. (2018). *Discovering statistics using IBM SPSS statistics* (5th ed.). SAGE Publications.

[cit0022] Frank, C. R. , Xiang, X. , Stagg, B. C. , & Ehrlich, J. R. (2019). Longitudinal associations of self-reported vision impairment with symptoms of anxiety and depression among older adults in the United States. *JAMA Ophthalmology* , *137* (7), 793. 10.1001/jamaophthalmol.2019.1085 31095253 PMC6537761

[cit0023] Ge, L. , Yap, C. W. , Ong, R. , & Heng, B. H. (2017). Social isolation, loneliness and their relationships with depressive symptoms: A population-based study. *PloS One* , *12* (8), e0182145. 10.1371/journal.pone.0182145 28832594 PMC5568112

[cit0024] Harper, M. (2012). Taming the amygdala: An EEG analysis of exposure therapy for the traumatized. *Traumatology (Tallahassee, Fla.)* , *18* (2), 61–74. 10.1177/1534765611429082

[cit0025] Heesterbeek, T. J. , Van Der Aa, H. P. A. , Van Rens, G. H. M. B. , Twisk, J. W. R. , & Van Nispen, R. M. A. (2017). The incidence and predictors of depressive and anxiety symptoms in older adults with vision impairment: A longitudinal prospective cohort study. *Ophthalmic and Physiological Optics* , *37* (4), 385–398. 10.1111/opo.12388 28516509

[cit0026] Holt-Lunstad, J. , Smith, T. B. , Baker, M. , Harris, T. , & Stephenson, D. (2015). Loneliness and social isolation as risk factors for mortality: A meta-analytic review. *Perspectives on Psychological Science* , *10* (2), 227–237. 10.1177/1745691614568352 25910392

[cit0027] Hornsveld, R. H. J. , Muris, P. , & Kraaimaat, F. W. (2011). The novaco anger Scale–Provocation inventory (1994 version) in Dutch forensic psychiatric patients. *Psychological Assessment* , *23* (4), 937–944. 10.1037/a0024018 21668125

[cit0028] IBM Corp . (2023). Released 2023, *IBM SPSS Statistics for Windows* . Armonk, NY: IBM Corp Version 29.0.2.0.

[cit0029] Khoo, K. , Man, R. E. K. , Rees, G. , Gupta, P. , Lamoureux, E. L. , & Fenwick, E. K. (2019). The relationship between diabetic retinopathy and psychosocial functioning: A systematic review. *Quality of Life Research* , *28* (8), 2017–2039. 10.1007/s11136-019-02165-1 30879245

[cit0030] Look, C. J. (2007). *Improve your eyesight naturally* . AuthorHouse.

[cit0031] Loprinzi, P. D. (2014). Influence of visual acuity on anxiety, panic and depression disorders among young and middle age adults in the United States. *Journal of Affective Disorders* , *167* , 8–11. 10.1016/j.jad.2014.05.052 25082107

[cit0032] Lundeen, E. A. , Saydah, S. , Ehrlich, J. R. , & Saaddine, J. (2022). Self-reported vision impairment and psychological distress in U.S. Adults. *Ophthalmic Epidemiology* , *29* (2), 171–181. 10.1080/09286586.2021.1918177 33896341 PMC10949979

[cit0033] Mangione, C. M. , Lee, P. P. , Gutierrez, P. R. , Spritzer, K. , Berry, S. , & Hays, R. D. (2001). Development of the 25-list-item national eye institute visual function questionnaire. *Archives of Ophthalmology* , *119* (7), 1050–1058. 10.1001/archopht.119.7.1050 11448327

[cit0034] Moher, D. , Schulz, K. F. , & Altman, D. G. & CONSORT. ( 2001). The CONSORT statement: Revised recommendations for improving the quality of reports of parallel group randomized trials. *BMC Medical Research Methodology* , *1* , 2. 10.1186/1471-2288-1-2 11336663 PMC32201

[cit0035] Mick, P. , Parfyonov, M. , Wittich, W. , Phillips, N. , Guthrie, D. , & Kathleen Pichora-Fuller, M. (2018). Associations between sensory loss and social networks, participation, support, and loneliness: Analysis of the Canadian Longitudinal Study on Aging. *Canadian family physician Medecin de famille canadien* , *64* (1), e33–e41. 29358266 PMC5962968

[cit0036] Nelms, J. A. , & Castel, L. (2016). A systematic review and meta-analysis of randomized and nonrandomized trials of clinical emotional freedom techniques (EFT) for the treatment of depression. *Explore (New York, N.Y.)* , *12* (6), 416–426. 10.1016/j.explore.2016.08.001 27843054

[cit0037] Novaco, R. W. (2003). *The Novaco anger scale and provocation inventory: NAS-PI* . Western Psychological Services.

[cit0038] Nyman, S. R. , Dibb, B. , Victor, C. R. , & Gosney, M. A. (2012). Emotional well-being and adjustment to vision loss in later life: A meta-synthesis of qualitative studies. *Disability and Rehabilitation* , *34* (12), 971–981. 10.3109/09638288.2011.626487 22066708

[cit0039] Orr, P. , Rentz, A. M. , Margolis, M. K. , Revicki, D. A. , Dolan, C. M. , Colman, S. , Fine, J. T. , & Bressler, N. M. (2011). Validation of The National eye institute visual function Questionnaire-25 (NEI VFQ-25) in age-related macular degeneration. *Investigative Opthalmology & Visual Science* , *52* (6), 3354. 10.1167/iovs.10-5645 21282568

[cit0040] Qualtrics . (2024). Qualtrics XM, *Computer Software* . Qualtrics. https://www.Qualtrics.com

[cit0041] Rees, G. , Ponczek, E. , Hassell, J. , Keeffe, J. E. , & Lamoureux, E. L. (2010). Psychological outcomes following interventions for people with low vision: A systematic review. *Expert Review of Ophthalmology* , *5* (3), 385–403. 10.1586/eop.10.32

[cit0042] Rohleder, N. (2019). Stress and inflammation – the need to address the gap in the transition between acute and chronic stress effects. *Psychoneuroendocrinology* , *105* , 164–171. 10.1016/j.psyneuen.2019.02.021 30826163

[cit0043] Burton, M. J. , Ramke, J. , Marques, A. P. , Bourne, R. R. A. , Congdon, N. , Jones, I. , Ah Tong, B. A. M. , Arunga, S. , Bachani, D. , Bascaran, C. , Bastawrous, A. , Blanchet, K. , Braithwaite, T. , Buchan, J. C. , Cairns, J. , Cama, A. , Chagunda, M. , Chuluunkhuu, C. , Cooper, A. , … Foster, A. (2021). The lancet global health commission on global eye health: Vision beyond 2020. *The Lancet Global Health* , *9* (4), e489–e551. 10.1016/S2214-109X(20)30488-5 33607016 PMC7966694

[cit0044] Sabel, B. A. , Wang, J. , Cárdenas-Morales, L. , Faiq, M. , & Heim, C. (2018). Mental stress as consequence and cause of vision loss: The Dawn of psychosomatic ophthalmology for preventive and personalized Medicine. *EPMA Journal* , *9* (2), 133–160. 10.1007/s13167-018-0136-8 29896314 PMC5972137

[cit0045] Simning, A. , Fox, M. L. , Barnett, S. L. , Sorensen, S. , & Conwell, Y. (2019). Depressive and anxiety symptoms in older adults with auditory, vision, and dual sensory impairment. *Journal of Aging and Health* , *31* (8), 1353–1375. 10.1177/0898264318781123 29896982 PMC6274614

[cit0046] Stapleton, P. , & Stewart, M. (2020). Comparison of the effectiveness of two modalities of group delivery of emotional freedom technique (EFT) intervention for food cravings: Online versus in-person. *Open Journal of Social Sciences* , *08* (02), 158–181. 10.4236/jss.2020.82014

[cit0047] Stapleton, P. , & Janzen, N. (2025). *The evidence based EFT manual: Foundations, key concepts, and core techniques of clinical EFT* . Imprint.

[cit0048] Tabachnick, B. G. , & Fidell, L. S. (2019). *Using multivariate statistics (Seventh edition).* Pearson.

[cit0049] Teitelman, J. , & Copolillo, A. (2005). Psychosocial issues in older adults’ adjustment to vision loss: Findings from qualitative interviews and focus groups. *The American Journal of Occupational Therapy* , *59* (4), 409–417. 10.5014/ajot.59.4.409 16124207

[cit0050] Tomitaka, S. (2020). Patterns of item score and total score distributions on depression rating scales in the general population: Evidence and mechanisms. *Heliyon* , *6* (12. e05862. 10.1016/j.heliyon.2020.e05862 33426345 PMC7777072

[cit0051] van der Aa, H. P. A. , Comijs, H. C. , Penninx, B. W. J. H. , van Rens, G. H. M. B. , & van Nispen, R. M. A. (2015). Major depressive and anxiety disorders in visually impaired older adults. *Investigative Ophthalmology & Visual Science* , *56* (2), 849–854. 10.1167/iovs.14-15848 25604690

[cit0052] van der Aa, H. P. A. , Margrain, T. H. , van Rens, G. H. M. B. , Heymans, M. W. , & van Nispen, R. M. A. (2016). Psychosocial interventions to improve mental health in adults with vision impairment: Systematic review and meta-analysis. *Ophthalmic and Physiological Optics* , *36* (5), 584–606. 10.1111/opo.12313 27580757

[cit0053] van Nispen, R. M. A. , Vreeken, H. L. , Comijs, H. C. , Deeg, D. J. H. , & van Rens, G. H. M. B. (2016). Role of vision loss, functional limitations and the supporting network in depression in a general population. *Acta Ophthalmologica* , *94* (1), 76–82. 10.1111/aos.12896 26545339

[cit0054] Wolpe, J. (1973). *The practice of behaviour therapy* (2nd ed.). New York, NY: Pergamon.

[cit0055] World Health Organization . (2019). *World report on vision* . https://www.who.int/publications/i/item/9789241516570

[cit0056] Xiang, X. , Freedman, V. A. , Shah, K. , Hu, R. X. , Stagg, B. C. , & Ehrlich, J. R. (2020). Self-reported vision impairment and subjective well-being in older adults: A longitudinal mediation analysis. *The Journals of Gerontology: Series A* , *75* (3), 589–595. 10.1093/gerona/glz148 PMC7328199 31169894

[cit0057] Zigmond, A. S. , & Snaith, R. P. (1983). The hospital anxiety and depression scale. *Acta Psychiatrica Scandinavica* , *67* (6), 361–370. 10.1111/j.1600-0447.1983.tb09716.x 6880820

