# Supplementary material for: Clinical EFT for individuals with visual impairment: effects on psychological wellbeing and subjective visual functioning
Source: Health Psychol Behav Med. 2026 Jul 7;14(1):2699488. doi: 10.1080/21642850.2026.2699488 (PMC13348127; doi:10.1080/21642850.2026.2699488)
Supplement: Supplementary Material — ITT_Analysis_Eyesight_EFT_SupplementaryCleanVersion.docx [file RHPB_A_2699488_SM8663.docx]

**Clinical EFT for Visually Impaired Individuals: Effects on Psychological Wellbeing and Subjective Visual Functioning**

**Health Psychology and Behavioral Medicine**

**Intention to Treat Analysis**

The analyses were conducted according to the intention-to-treat (ITT) principle, whereby all randomized participants were included in the analyses regardless of treatment adherence or completion status. To maximise use of available data and minimise bias associated with participant attrition, linear mixed-effects models were employed rather than complete-case analyses or last-observation-carried-forward procedures.

Mixed models included fixed effects for treatment group, time, and the group-by-time interaction, with participant included as a random effect to account for within-subject correlation across repeated assessments. An unstructured covariance matrix was specified for repeated observations. Parameters were estimated using restricted maximum likelihood (REML).

This approach is consistent with contemporary recommendations for randomized controlled trials because it utilizes all available observations without requiring imputation of missing outcome data and provides unbiased estimates under the assumption that data are missing at random (MAR). The primary test of treatment efficacy was the Group × Time interaction, which evaluated whether changes over time differed significantly between the EFT intervention and waitlist control conditions.

Sensitivity analyses using multiple imputation (50 imputations) produced substantively identical conclusions and are available in the Supplementary Materials.

**Complete summary: Intention-to-Treat Analyses**

Intention-to-treat analyses were conducted using linear mixed-effects models estimated with restricted maximum likelihood. Significant Group × Time interactions were observed across all outcome measures, indicating greater improvements among participants receiving EFT relative to waitlist controls. Significant treatment effects were observed for anxiety, F(1, 307.76) = 26.94, p < .001, depression, F(1, 304.93) = 33.47, p < .001, anger, F(1, 310.81) = 32.45, p < .001, and vision functioning, F(1, 300.90) = 25.05, p < .001.

Estimated marginal means demonstrated minimal change during the waitlist period, whereas participants receiving EFT exhibited substantial improvements. Anxiety decreased from 8.31 to 6.34 in the EFT group compared with 8.70 to 8.40 in the waitlist group. Depression decreased from 5.31 to 3.93 following EFT while increasing slightly in the waitlist group (5.42 to 5.73). Anger decreased from 44.90 to 36.50 following EFT compared with negligible change in the waitlist condition (44.75 to 44.24). Vision functioning improved from 82.86 to 87.77 following EFT while remaining stable in the waitlist group (81.82 to 81.61). Detailed model estimates are provided in the Supplementary Materials.

Longitudinal intention-to-treat mixed-effects analyses were conducted to examine maintenance of treatment gains following EFT. Significant effects of time were observed for anxiety, F(3, 358.74) = 27.36, p < .001, depression, F(3, 344.38) = 27.81, p < .001, anger, F(3, 367.01) = 36.21, p < .001, and vision functioning, F(3, 343.17) = 27.71, p < .001. Bonferroni-adjusted pairwise comparisons indicated significant improvements from pretreatment to post-treatment across all outcomes (all p < .001). Improvements were maintained at both 3- and 6-month follow-up assessments. Anxiety, anger, and vision functioning remained significantly improved relative to pretreatment at both follow-up assessments (all p < .001), while depression remained significantly improved at 3 months (p < .001) and 6 months (p = .004). No significant differences were observed between post-treatment and either follow-up assessment for any outcome (all p > .95), indicating sustained maintenance of treatment gains over the six-month follow-up period.

**Pre to Post**

**Anxiety**


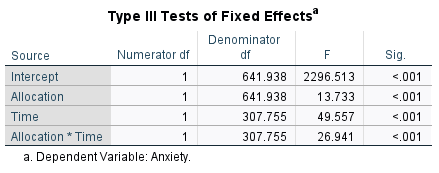


| **Allocation * Time^a^** | | | | | | |
| --- | --- | --- | --- | --- | --- | --- |
| Allocation | Time | Mean | Std. Error | df | 95% Confidence Interval | |
|  |  |  |  |  | Lower Bound | Upper Bound |
| Waitlist | Baseline | 8.700 | .244 | 654.042 | 8.221 | 9.180 |
|  | 8 Weeks | 8.402 | .277 | 835.159 | 7.858 | 8.946 |
| EFT | Baseline | 8.312 | .223 | 654.042 | 7.874 | 8.749 |
|  | 8 Weeks | 6.335 | .293 | 828.286 | 5.761 | 6.910 |
| a. Dependent Variable: Anxiety. | | | | | | |

An intention-to-treat mixed-effects analysis demonstrated a significant Group × Time interaction for anxiety, F(1, 307.76) = 26.94, p < .001. Estimated marginal means indicated that anxiety scores remained relatively stable in the waitlist condition (Baseline M = 8.70, 95% CI [8.22, 9.18]; 8 Weeks M = 8.40, 95% CI [7.86, 8.95]), whereas participants receiving EFT showed a substantial reduction in anxiety symptoms (Baseline M = 8.31, 95% CI [7.87, 8.75]; 8 Weeks M = 6.34, 95% CI [5.76, 6.91]). These findings indicate significantly greater reductions in anxiety among participants receiving EFT compared with those assigned to the waitlist control condition.

**Depression**

| **Type III Tests of Fixed Effects^a^** | | | | |
| --- | --- | --- | --- | --- |
| Source | Numerator df | Denominator df | F | Sig. |
| Intercept | 1 | 638.722 | 1259.315 | <.001 |
| Allocation | 1 | 638.722 | 11.135 | <.001 |
| Time | 1 | 304.925 | 13.733 | <.001 |
| Allocation * Time | 1 | 304.925 | 33.466 | <.001 |
| a. Dependent Variable: Depression. | | | | |

| **Allocation * Time^a^** | | | | | | |
| --- | --- | --- | --- | --- | --- | --- |
| Allocation | Time | Mean | Std. Error | df | 95% Confidence Interval | |
|  |  |  |  |  | Lower Bound | Upper Bound |
| Waitlist | Baseline | 5.423 | .212 | 655.604 | 5.007 | 5.839 |
|  | 8 Weeks | 5.726 | .242 | 840.964 | 5.250 | 6.202 |
| EFT | Baseline | 5.308 | .193 | 655.604 | 4.929 | 5.688 |
|  | 8 Weeks | 3.925 | .258 | 818.604 | 3.418 | 4.431 |
| a. Dependent Variable: Depression. | | | | | | |

An intention-to-treat mixed-effects analysis demonstrated a significant Group × Time interaction for depression, F(1, 304.93) = 33.47, p < .001. Estimated marginal means indicated that depressive symptoms increased slightly in the waitlist condition (Baseline M = 5.42, 95% CI [5.01, 5.84]; 8 Weeks M = 5.73, 95% CI [5.25, 6.20]), whereas participants receiving EFT demonstrated a reduction in depressive symptoms (Baseline M = 5.31, 95% CI [4.93, 5.69]; 8 Weeks M = 3.93, 95% CI [3.42, 4.43]). These findings indicate significantly greater reductions in depression among participants receiving EFT compared with those assigned to the waitlist control condition.

NAS Anger

| **Type III Tests of Fixed Effects^a^** | | | | |
| --- | --- | --- | --- | --- |
| Source | Numerator df | Denominator df | F | Sig. |
| Intercept | 1 | 641.553 | 4063.586 | <.001 |
| Allocation | 1 | 641.553 | 8.037 | .005 |
| Time | 1 | 310.814 | 41.301 | <.001 |
| Allocation * Time | 1 | 310.814 | 32.448 | <.001 |
| a. Dependent Variable: NASScore. | | | | |

| **Allocation * Time^a^** | | | | | | |
| --- | --- | --- | --- | --- | --- | --- |
| Allocation | Time | Mean | Std. Error | df | 95% Confidence Interval | |
|  |  |  |  |  | Lower Bound | Upper Bound |
| Waitlist | Baseline | 44.745 | .984 | 660.911 | 42.813 | 46.678 |
|  | 8 Weeks | 44.239 | 1.134 | 842.273 | 42.013 | 46.466 |
| EFT | Baseline | 44.903 | .901 | 660.911 | 43.135 | 46.671 |
|  | 8 Weeks | 36.504 | 1.211 | 813.486 | 34.128 | 38.881 |
| a. Dependent Variable: NASScore. | | | | | | |

An intention-to-treat mixed-effects analysis demonstrated a significant Group × Time interaction for anger, F(1, 310.81) = 32.45, p < .001. Estimated marginal means indicated that anger levels remained largely unchanged in the waitlist condition (Baseline M = 44.75, 95% CI [42.81, 46.68]; 8 Weeks M = 44.24, 95% CI [42.01, 46.47]), whereas participants receiving EFT demonstrated a substantial reduction in anger (Baseline M = 44.90, 95% CI [43.14, 46.67]; 8 Weeks M = 36.50, 95% CI [34.13, 38.88]). These findings indicate significantly greater reductions in anger among participants receiving EFT compared with those assigned to the waitlist control condition.

**VFQ**

| **Type III Tests of Fixed Effects^a^** | | | | |
| --- | --- | --- | --- | --- |
| Source | Numerator df | Denominator df | F | Sig. |
| Intercept | 1 | 632.739 | 23742.040 | <.001 |
| Allocation | 1 | 632.739 | 11.048 | <.001 |
| Time | 1 | 300.901 | 21.210 | <.001 |
| Allocation * Time | 1 | 300.901 | 25.054 | <.001 |
| a. Dependent Variable: VFQ. | | | | |

| **Allocation * Time^a^** | | | | | | |
| --- | --- | --- | --- | --- | --- | --- |
| Allocation | Time | Mean | Std. Error | df | 95% Confidence Interval | |
|  |  |  |  |  | Lower Bound | Upper Bound |
| Waitlist | Baseline | 81.815 | .798 | 642.499 | 80.248 | 83.382 |
|  | 8 Weeks | 81.611 | .900 | 824.171 | 79.843 | 83.378 |
| EFT | Baseline | 82.862 | .730 | 642.499 | 81.429 | 84.294 |
|  | 8 Weeks | 87.770 | .945 | 831.825 | 85.916 | 89.624 |
| a. Dependent Variable: VFQ. | | | | | | |

Vision functioning remained unchanged during the wait period (-0.20).

Participants receiving EFT improved by approximately 4.91 points.

The between-group difference in change was approximately 5.11 points.

**Summary**

| **Outcome** | **F(df)** | **p** |
| --- | --- | --- |
| HADS Anxiety | F(1, 307.76) = 26.94 | < .001 |
| HADS Depression | F(1, 304.93) = 33.47 | < .001 |
| NAS | F(1, 310.81) = 32.45 | < .001 |
| VFQ Composite | F(1, 300.90) = 25.05 | < .001 |

Intention-to-treat analyses were conducted using linear mixed-effects models with restricted maximum likelihood estimation. Significant Group × Time interactions were observed across all outcome measures. For anxiety, participants receiving EFT demonstrated significantly greater reductions than participants assigned to the waitlist condition, F(1, 307.76) = 26.94, p < .001. Similarly, significant Group × Time interactions were observed for depression, F(1, 304.93) = 33.47, p < .001, and anger, F(1, 310.81) = 32.45, p < .001, indicating greater improvements among participants receiving EFT relative to waitlist controls. Vision functioning also improved significantly following EFT compared with the waitlist condition, F(1, 300.90) = 25.05, p < .001.

Estimated marginal means demonstrated minimal change during the waitlist period across anxiety, anger, and vision functioning outcomes, whereas participants receiving EFT exhibited clinically meaningful improvements. Anxiety decreased from 8.31 to 6.34 in the EFT group compared with 8.70 to 8.40 in the waitlist group. Depression decreased from 5.31 to 3.93 following EFT while slightly increasing in the waitlist group (5.42 to 5.73). Anger decreased from 44.90 to 36.50 following EFT compared with negligible change in the waitlist condition (44.75 to 44.24). Vision functioning improved from 82.86 to 87.77 following EFT while remaining stable in the waitlist group (81.82 to 81.61).

All primary analyses were conducted according to the intention-to-treat principle using linear mixed-effects models estimated with restricted maximum likelihood (REML). The randomized comparison evaluated change over the initial 8-week intervention period, comparing participants allocated to immediate EFT treatment (Pre to Post) with participants allocated to the waitlist control condition (Pre to Pre2). Fixed effects included group, time, and the Group × Time interaction, with participant included as a random effect. Mixed-effects models were selected because they use all available observations from randomized participants and provide valid estimates under a missing-at-random assumption without requiring listwise deletion of incomplete cases.

**Follow up**

| **Type III Tests of Fixed Effects^a^** | | | | |
| --- | --- | --- | --- | --- |
| Source | Numerator df | Denominator df | F | Sig. |
| Intercept | 1 | 726.300 | 1225.961 | <.001 |
| Time | 3 | 358.740 | 27.355 | <.001 |
| a. Dependent Variable: Anxiety. | | | | |

| **Estimates^a^** | | | | | |
| --- | --- | --- | --- | --- | --- |
| Time | Mean | Std. Error | df | 95% Confidence Interval | |
|  |  |  |  | Lower Bound | Upper Bound |
| PreTreatment | 8.276 | .179 | 565.747 | 7.925 | 8.627 |
| PostTreatment | 6.645 | .227 | 804.553 | 6.199 | 7.092 |
| 3Month | 6.798 | .318 | 632.565 | 6.175 | 7.422 |
| 6Month | 6.706 | .373 | 542.814 | 5.974 | 7.439 |
| a. Dependent Variable: Anxiety. | | | | | |

| **Pairwise Comparisons^a^** | | | | | | | |
| --- | --- | --- | --- | --- | --- | --- | --- |
| (I) Time | (J) Time | Mean Difference (I-J) | Std. Error | df | Sig.^c^ | 95% Confidence Interval for Difference^c^ | |
|  |  |  |  |  |  | Lower Bound | Upper Bound |
| PreTreatment | PostTreatment | 1.631^*^ | .195 | 378.177 | <.001 | 1.113 | 2.148 |
|  | 3Month | 1.478^*^ | .294 | 365.119 | <.001 | .697 | 2.259 |
|  | 6Month | 1.569^*^ | .354 | 359.487 | <.001 | .631 | 2.508 |
| PostTreatment | PreTreatment | -1.631^*^ | .195 | 378.177 | <.001 | -2.148 | -1.113 |
|  | 3Month | -.153 | .306 | 346.509 | 1.000 | -.964 | .658 |
|  | 6Month | -.061 | .361 | 345.066 | 1.000 | -1.020 | .897 |
| 3Month | PreTreatment | -1.478^*^ | .294 | 365.119 | <.001 | -2.259 | -.697 |
|  | PostTreatment | .153 | .306 | 346.509 | 1.000 | -.658 | .964 |
|  | 6Month | .092 | .402 | 334.882 | 1.000 | -.974 | 1.158 |
| 6Month | PreTreatment | -1.569^*^ | .354 | 359.487 | <.001 | -2.508 | -.631 |
|  | PostTreatment | .061 | .361 | 345.066 | 1.000 | -.897 | 1.020 |
|  | 3Month | -.092 | .402 | 334.882 | 1.000 | -1.158 | .974 |
| Based on estimated marginal means | | | | | | | |
| *. The mean difference is significant at the .05 level. | | | | | | | |
| a. Dependent Variable: Anxiety. | | | | | | | |
| c. Adjustment for multiple comparisons: Bonferroni. | | | | | | | |

A longitudinal intention-to-treat mixed-effects analysis demonstrated a significant effect of time for anxiety, F(3, 358.74) = 27.36, p < .001. Pairwise comparisons with Bonferroni adjustment indicated significant reductions in anxiety from pre-treatment (M = 8.28) to post-treatment (M = 6.65), p < .001. Improvements were maintained at both 3 months (M = 6.80) and 6 months (M = 6.71), with anxiety remaining significantly lower than pretreatment levels at both follow-up assessments (both p < .001). No significant differences were observed between post-treatment and either 3-month or 6-month follow-up assessments (both p = 1.00), indicating maintenance of treatment gains over time.

**Depression**

| **Type III Tests of Fixed Effects^a^** | | | | |
| --- | --- | --- | --- | --- |
| Source | Numerator df | Denominator df | F | Sig. |
| Intercept | 1 | 714.492 | 627.082 | <.001 |
| Time | 3 | 344.375 | 27.813 | <.001 |
| a. Dependent Variable: Depression. | | | | |

| **Estimates^a^** | | | | | |
| --- | --- | --- | --- | --- | --- |
| Time | Mean | Std. Error | df | 95% Confidence Interval | |
|  |  |  |  | Lower Bound | Upper Bound |
| PreTreatment | 5.450 | .160 | 548.949 | 5.135 | 5.765 |
| PostTreatment | 3.964 | .201 | 802.060 | 3.568 | 4.359 |
| 3Month | 4.309 | .278 | 638.069 | 3.763 | 4.854 |
| 6Month | 4.399 | .325 | 543.084 | 3.761 | 5.037 |
| a. Dependent Variable: Depression. | | | | | |

| **Pairwise Comparisons^a^** | | | | | | | |
| --- | --- | --- | --- | --- | --- | --- | --- |
| (I) Time | (J) Time | Mean Difference (I-J) | Std. Error | df | Sig.^c^ | 95% Confidence Interval for Difference^c^ | |
|  |  |  |  |  |  | Lower Bound | Upper Bound |
| PreTreatment | PostTreatment | 1.486^*^ | .168 | 362.383 | <.001 | 1.039 | 1.933 |
|  | 3Month | 1.141^*^ | .254 | 349.972 | <.001 | .468 | 1.815 |
|  | 6Month | 1.051^*^ | .305 | 344.838 | .004 | .241 | 1.860 |
| PostTreatment | PreTreatment | -1.486^*^ | .168 | 362.383 | <.001 | -1.933 | -1.039 |
|  | 3Month | -.345 | .263 | 333.205 | 1.000 | -1.044 | .354 |
|  | 6Month | -.436 | .311 | 331.848 | .976 | -1.262 | .391 |
| 3Month | PreTreatment | -1.141^*^ | .254 | 349.972 | <.001 | -1.815 | -.468 |
|  | PostTreatment | .345 | .263 | 333.205 | 1.000 | -.354 | 1.044 |
|  | 6Month | -.091 | .346 | 322.817 | 1.000 | -1.009 | .827 |
| 6Month | PreTreatment | -1.051^*^ | .305 | 344.838 | .004 | -1.860 | -.241 |
|  | PostTreatment | .436 | .311 | 331.848 | .976 | -.391 | 1.262 |
|  | 3Month | .091 | .346 | 322.817 | 1.000 | -.827 | 1.009 |
| Based on estimated marginal means | | | | | | | |
| *. The mean difference is significant at the .05 level. | | | | | | | |
| a. Dependent Variable: Depression. | | | | | | | |
| c. Adjustment for multiple comparisons: Bonferroni. | | | | | | | |

A longitudinal intention-to-treat mixed-effects analysis demonstrated a significant effect of time for depression, F(3, 344.38) = 27.81, p < .001. Pairwise comparisons with Bonferroni adjustment indicated significant reductions in depression from pre-treatment (M = 5.45) to post-treatment (M = 3.96), p < .001. Improvements were maintained at both 3 months (M = 4.31) and 6 months (M = 4.40), with depression remaining significantly lower than pretreatment levels at both follow-up assessments (p < .001 and p = .004, respectively). No significant differences were observed between post-treatment and either follow-up assessment (both p > .95), indicating maintenance of treatment gains over time.

**Anger**

| **Type III Tests of Fixed Effects^a^** | | | | |
| --- | --- | --- | --- | --- |
| Source | Numerator df | Denominator df | F | Sig. |
| Intercept | 1 | 730.261 | 2171.814 | <.001 |
| Time | 3 | 367.014 | 36.212 | <.001 |
| a. Dependent Variable: NASScore. | | | | |

| **Estimates^a^** | | | | | |
| --- | --- | --- | --- | --- | --- |
| Time | Mean | Std. Error | df | 95% Confidence Interval | |
|  |  |  |  | Lower Bound | Upper Bound |
| PreTreatment | 44.356 | .720 | 575.065 | 42.941 | 45.771 |
| PostTreatment | 36.605 | .934 | 800.852 | 34.772 | 38.438 |
| 3Month | 36.458 | 1.330 | 618.071 | 33.847 | 39.069 |
| 6Month | 36.698 | 1.561 | 536.199 | 33.630 | 39.765 |
| a. Dependent Variable: NASScore. | | | | | |

| **Pairwise Comparisons^a^** | | | | | | | |
| --- | --- | --- | --- | --- | --- | --- | --- |
| (I) Time | (J) Time | Mean Difference (I-J) | Std. Error | df | Sig.^c^ | 95% Confidence Interval for Difference^c^ | |
|  |  |  |  |  |  | Lower Bound | Upper Bound |
| PreTreatment | PostTreatment | 7.751^*^ | .826 | 387.613 | <.001 | 5.561 | 9.942 |
|  | 3Month | 7.899^*^ | 1.253 | 374.689 | <.001 | 4.575 | 11.223 |
|  | 6Month | 7.659^*^ | 1.498 | 368.871 | <.001 | 3.685 | 11.632 |
| PostTreatment | PreTreatment | -7.751^*^ | .826 | 387.613 | <.001 | -9.942 | -5.561 |
|  | 3Month | .147 | 1.300 | 353.104 | 1.000 | -3.303 | 3.598 |
|  | 6Month | -.093 | 1.532 | 352.263 | 1.000 | -4.158 | 3.972 |
| 3Month | PreTreatment | -7.899^*^ | 1.253 | 374.689 | <.001 | -11.223 | -4.575 |
|  | PostTreatment | -.147 | 1.300 | 353.104 | 1.000 | -3.598 | 3.303 |
|  | 6Month | -.240 | 1.708 | 340.260 | 1.000 | -4.772 | 4.292 |
| 6Month | PreTreatment | -7.659^*^ | 1.498 | 368.871 | <.001 | -11.632 | -3.685 |
|  | PostTreatment | .093 | 1.532 | 352.263 | 1.000 | -3.972 | 4.158 |
|  | 3Month | .240 | 1.708 | 340.260 | 1.000 | -4.292 | 4.772 |
| Based on estimated marginal means | | | | | | | |
| *. The mean difference is significant at the .05 level. | | | | | | | |
| a. Dependent Variable: NASScore. | | | | | | | |
| c. Adjustment for multiple comparisons: Bonferroni. | | | | | | | |

A longitudinal intention-to-treat mixed-effects analysis demonstrated a significant effect of time for anger, F(3, 367.01) = 36.21, p < .001. Pairwise comparisons with Bonferroni adjustment indicated significant reductions in anger from pre-treatment (M = 44.36) to post-treatment (M = 36.61), p < .001. Improvements were maintained at both 3 months (M = 36.46) and 6 months (M = 36.70), with anger remaining significantly lower than pretreatment levels at both follow-up assessments (both p < .001). No significant differences were observed between post-treatment and either 3-month or 6-month follow-up assessments (both p = 1.00), indicating sustained maintenance of treatment gains over time.

**VFQ**

| **Type III Tests of Fixed Effects^a^** | | | | |
| --- | --- | --- | --- | --- |
| Source | Numerator df | Denominator df | F | Sig. |
| Intercept | 1 | 704.687 | 17036.034 | <.001 |
| Time | 3 | 343.167 | 27.710 | <.001 |
| a. Dependent Variable: VFQ. | | | | |

| **Estimates^a^** | | | | | |
| --- | --- | --- | --- | --- | --- |
| Time | Mean | Std. Error | df | 95% Confidence Interval | |
|  |  |  |  | Lower Bound | Upper Bound |
| PreTreatment | 82.403 | .587 | 542.791 | 81.251 | 83.556 |
| PostTreatment | 87.296 | .734 | 792.907 | 85.855 | 88.737 |
| 3Month | 87.823 | 1.006 | 639.902 | 85.848 | 89.798 |
| 6Month | 87.210 | 1.169 | 548.332 | 84.915 | 89.506 |
| a. Dependent Variable: VFQ. | | | | | |

| **Pairwise Comparisons^a^** | | | | | | | |
| --- | --- | --- | --- | --- | --- | --- | --- |
| (I) Time | (J) Time | Mean Difference (I-J) | Std. Error | df | Sig.^c^ | 95% Confidence Interval for Difference^c^ | |
|  |  |  |  |  |  | Lower Bound | Upper Bound |
| PreTreatment | PostTreatment | -4.893^*^ | .606 | 360.308 | <.001 | -6.502 | -3.284 |
|  | 3Month | -5.420^*^ | .914 | 348.614 | <.001 | -7.846 | -2.994 |
|  | 6Month | -4.807^*^ | 1.092 | 344.021 | <.001 | -7.704 | -1.910 |
| PostTreatment | PreTreatment | 4.893^*^ | .606 | 360.308 | <.001 | 3.284 | 6.502 |
|  | 3Month | -.527 | .946 | 332.044 | 1.000 | -3.038 | 1.983 |
|  | 6Month | .086 | 1.114 | 331.269 | 1.000 | -2.871 | 3.042 |
| 3Month | PreTreatment | 5.420^*^ | .914 | 348.614 | <.001 | 2.994 | 7.846 |
|  | PostTreatment | .527 | .946 | 332.044 | 1.000 | -1.983 | 3.038 |
|  | 6Month | .613 | 1.239 | 322.670 | 1.000 | -2.675 | 3.901 |
| 6Month | PreTreatment | 4.807^*^ | 1.092 | 344.021 | <.001 | 1.910 | 7.704 |
|  | PostTreatment | -.086 | 1.114 | 331.269 | 1.000 | -3.042 | 2.871 |
|  | 3Month | -.613 | 1.239 | 322.670 | 1.000 | -3.901 | 2.675 |
| Based on estimated marginal means | | | | | | | |
| *. The mean difference is significant at the .05 level. | | | | | | | |
| a. Dependent Variable: VFQ. | | | | | | | |
| c. Adjustment for multiple comparisons: Bonferroni. | | | | | | | |
